# Increased Work Experience Associated with Less Stigmatizing Attitudes towards People Living with HIV among Thai Healthcare Personnel

**DOI:** 10.3390/ijerph18189830

**Published:** 2021-09-18

**Authors:** Kriengkrai Srithanaviboonchai, Porntip Khemngern, Jarun Chuayen, Taweesap Siraprapasiri

**Affiliations:** 1 Faculty of Medicine, Chiang Mai University, 110 Intavaroros, Sriphum, Muang, Chiang Mai 50200, Thailand; 2 Research Institute for Health Sciences, Chiang Mai University, 110 Intavaroros, Sriphum, Muang, Chiang Mai 50200, Thailand; nui.jk17635@gmail.com; 3 Department of Disease Control, Ministry of Public Health, 88/21 Tiwanon Road, Thaladkwan, Muang, Nonthaburee 11000, Thailand; itimpornt@yahoo.com (P.K.); taweesap@rocketmail.com (T.S.)

**Keywords:** HIV-related stigma, healthcare personnel, negative attitude, Thailand

## Abstract

HIV-related stigma in health facilities has been suggested as a primary target for HIV-related stigma reduction. The objective of this study was to describe negative attitudes among Thai healthcare personnel (HCP) toward PLHIV. This nationwide probability sampled survey was conducted in 2019 in 12 provinces in Thailand and Bangkok, the capital. Participants were considered to have stigmatizing attitudes toward PLHIV if they had a stigmatizing view in response to at least one of the four questions. Eighty-two percent of the 3056 respondents had at least one stigmatizing attitude. Younger HCP, ages < 30 (AOR = 1.60; 95%CI: 1.18–2.18) and 30–39 (AOR = 1.60; 95%CI: 1.21–2.12) were more likely to have stigmatizing attitudes towards PLHIV compared to those aged 50 and older. Being support staff, support-clinical (AOR = 1.89; 95%CI: 1.44–2.49) and support-nonclinical (AOR = 1.71; 95%CI: 1.24–2.36) as opposed to professional staff also increased the likelihood of having stigmatizing attitudes. Stigma was also more likely to be present in HCPs who did not work at HIV-focused clinics (AOR = 1.97; 95%CI: 1.57–2.48). HCP who had more work experience, especially related to PLHIV care, were less likely to have stigmatizing attitudes. These personnel could be good peer educators or role models for a stigma reduction campaign within their healthcare facilities.

## 1. Introduction

HIV-related stigma has been widely cited as a major obstacle in reaching global targets to ending the AIDS epidemic [1,2,3,4]. The United Nations Joint Program of HIV/AIDS (UNAIDS) defines HIV-related stigma and discrimination as: “a ‘process of devaluation’ of people either living with or associated with HIV and AIDS Discrimination follows stigma and is the unfair and unjust treatment of an individual based on his or her real or perceived HIV status” [5]. While PLHIV face discrimination in many areas of their lives, HIV-related stigma associated with health facilities is particularly concerning as these venues play a crucial role in ending the AIDS epidemic. Consequently, health facilities have been identified as primary targets for HIV-related stigma reduction [6].

Reported discriminatory practices by healthcare personnel (HCP) toward PLHIV and those perceived to be HIV-infected included humiliation and blame [7], refusal of care [7,8,9], physically labeling clients as HIV-positive [9], unnecessary referrals to other healthcare facilities [9], excessive precautions and physical distancing [7,9,10], suboptimal care [7], isolating patients for no sound medical reason [8], and testing for HIV or disclosing HIV status without consent [9,10].

HIV-related stigma in healthcare settings compromises the effectiveness of HIV prevention and control programs in many ways. Research has found that persons who engaged in HIV risk behaviors were reluctant to take HIV tests and other preventive measures due to anticipation of discriminatory practices at health facilities [11,12,13]. Among known HIV-infected individuals, many have avoided or delayed antiretroviral treatment (ART) if they anticipated or perceived discriminatory practices at health facilities [7,14,15]. HIV-infected patients in care who faced discrimination from HCP were more likely to be lost to follow-up, possibly leading to poor medical adherence and virologic failure [7,16,17].

There are three main causes of HIV-related stigma in health facilities: lack of awareness of what constitutes stigma and why it is problematic; fear of HIV infection due to incorrect knowledge about HIV transmission; and negative attitudes toward people living with HIV (PLHIV) [6]. They are recognized as actionable drivers of HIV-related stigma as they could be mitigated through proper interventions.

Of the three drivers of HIV-related stigma in health facilities, negative attitudes toward PLHIV are noteworthy and need to be emphasized. While lack of awareness of HIV-related stigma and unjust fear of HIV infection might simply be addressed through education and training, HCP negative attitudes toward PLHIV is more difficult to address. Socially, people associate HIV infection with behaviors perceived to be immoral or improper, namely promiscuous sex and illicit drugs use [18,19,20]. Several recent studies conducted in a variety of social and cultural contexts consistently described relatively high levels of stigmatizing attitudes toward PLHIV among healthcare providers [9,19,20,21,22,23,24].

Thailand has been admired by the international community for its achievements in tackling HIV/AIDS [25]. However, recent studies revealed that there are ongoing localized HIV epidemics in some key populations, especially men who have sex with men and transgender women [26,27,28,29]. In 2019, only 80% of people diagnosed with HIV received ART, below the national target of 90% [30]. This rate might be explained in part by HIV-related stigma in Thai health facilities. Information on the details, extent, and determinants of stigmatization is needed to properly design targeted interventions for the country.

The literature on HIV-related stigma in Thai health facilities is scarce and outdated according to a recent published review article [31]. The Stigma Index Survey conducted in 2009 by the Thai Network of People Living with HIV/AIDS found that around 20% of PLHIV respondents had been refused health or dental care [32]. Burmese migrant workers who were also HIV infected reported discrimination from Thai HCP when seeking care and treatment due to their migrant status [33]. A study conducted among student nurses in Bangkok found that PLHIV were stigmatized but were significantly less stigmatized than intravenous drug users [34]. Thus far, there has been no research directly addressing stigmatizing attitudes toward PLHIV among Thai HCP.

As part of systematic efforts to measure and reduce HIV-related stigma [35], the country has established a national surveillance system to monitor HIV-related stigma in government health facilities. The tools used in this surveillance have been adapted from recommended global tools. The process of developing and standardizing these tools has been published elsewhere [36]. The objectives of this study were to quantify and describe negative attitudes among Thai HCP toward PLHIV as well as to identify the determinants of these negative attitudes using the data from the 2019 Thai national surveillance survey.

## 2. Materials and Methods

### 2.1. Study Setting and Selection of Study Sites

This cross-sectional survey study was conducted in 2019 as the core activity of Thailand’s national surveillance system to monitor HIV-related stigma in government healthcare facilities. All provinces including Bangkok were eligible to be included in the study. Thirteen geographical areas, 12 provincial centers of Thailand’s 12 health regions, plus the capital city of Bangkok, were then selected as the national surveillance sites. Apart from Bangkok, all provinces were purposively selected as the economic centers of each region and based on high HIV burden. The provinces represented were Chiang Rai, Phitsanulok, Nakhon Sawan, Saraburi, Nakorn Pathom, Chon Buri, Khon Kaen, Udon Thani, Nakorn Ratchasima, Ubon Ratchathani, Nakorn Si Thammarat, and Songkhla. The only province that was not the official center of its region was Chiang Rai, which was selected in place of Chiang Mai due to management issues. Within each province, all hospitals under the Office of Permanent Secretary, Ministry of Public Health, with HIV clinics were invited to participate in the survey. These included all regional, general, and community hospitals providing coverage for most of registered patients with PLHIV in their respective areas. For Bangkok, all hospitals under the Bangkok Metropolitan Administration were included in the study.

### 2.2. Study Population

All HCP who worked at these hospitals and provided direct services to patients and their relatives (regardless of patient HIV status) were eligible to participate in the study. These included both clinical and non-clinical staff. Staff who did not have direct contact with patients or their relatives (back-office staff), such as procurement, document, and personnel division staff were not eligible. The intention was to capture the perspectives of diverse hospital staff who may discriminate against PLHIV in the healthcare facilities.

### 2.3. Sample Size Calculation and Sampling

The sample size for each study area (12 provinces and Bangkok) was computed to estimate the level of HIV-related stigma in healthcare facilities at the provincial level. This was to make sure that each province had its own meaningful information for use. The sample sizes were calculated using the prevalence of stigmatizing attitudes among Thai HCP from an unpublished pilot study (85.8%) based on an estimation of *p* (*p* = 0.858), at the 95% confidence level, and with a 10% acceptable error bound [37]. The final figures were increased by 10% to compensate for expected incomplete questionnaires. The sample sizes ranged from 182 (Chiang Rai) to 312 (Pitsanulok). The samples of all 13 study areas were then combined for a total study sample size (3056).

The sample size at each hospital was determined proportional to the number of healthcare staff of the whole province. The names of all eligible HCP at particular participating hospitals were compiled. Simple random sampling was used to draw up a list of potential participants who would be invited to participate in the survey.

### 2.4. Measurements

The survey included questions on participant demographics including gender, age, profession, geographical region, whether the participant worked at an HIV-focused clinic, and estimated number of PLHIV that the participants has provided care for during the last 12 months.

Professions were categorized into 3 groups: (1) Health professionals (medical doctor, dentist, nurse, pharmacist, laboratory technician, physical therapist, psychologist, social worker, and radiologist), (2) Support-clinical (nurse aide, dental assistant, pharmacy assistant, paramedic, health educator, hospital porter, laboratory assistant, and radiologic assistant), and (3) Support non-clinical staff (cashier, receptionist, cleaner, driver, waiter, administrative staff, security guard, and medical record staff).

The study sites (hospitals) were divided into 5 geographical regions: (1) northern (Chiang Rai and Phitsanulok), (2) central (Nakhon Sawan, Saraburi, Nakorn Pathom, and Chon Buri), (3) northeastern (Khon Kaen, Udon Thani, Nakorn Ratchasima, and Ubon Ratchathani), (4) southern (Nakorn Si Thammarat and Songkhla) and (5) Capitol (Bangkok).

Clinics focused on the following treatment areas were defined as HIV-focused clinics: antiretroviral (ARV) treatment, HIV counseling, antenatal care, sexually transmitted diseases, and tuberculosis. Participants working at these clinics were considered HIV-focused HCP.

Questions were adapted from global tools and standardized for the Thai context [32]. Four items were used to measure negative attitudes towards PLHIV: (1) “Women living with HIV should be allowed to have babies if they wish,” (2) “People get infected with HIV because they engage in irresponsible/immoral behaviors,” (3) “People living with HIV should be ashamed of their HIV status,” and (4) “Most people living with HIV do not care that they could infect other people.” The response choices were “Strongly agree”, “Agree”, “Disagree”, and “Strongly disagree”. The “Strongly agree” and “Agree” answers were grouped as “Agree”. The “Disagree” and “Strongly disagree” answers were grouped and “Disagree”. Participants who disagreed with question 1 or agreed to questions 2, 3, or 4 were considered to have negative attitudes toward PLHIV. The final composite variable of negative attitudes toward PLHIV was used as the main outcome of the study.

### 2.5. Data Collection

The survey was conducted from June to September 2019. The data collection team included local, provincial, and regional health officers. A centralized training was administered to the study team prior to the actual data collection. Potential participants who were randomly selected from eligible staff were asked to come to a hospital conference room, usually during lunchtime. For the hospitals that required large samples, these informational meetings were limited to 20 people at a time to allow participants to sit far apart to maintain privacy. Study staff informed potential participants about the objectives of the study, benefits and risks, and how long the questionnaire would take. Interested participants who were willing to participate provided signed informed consent, and then read and completed the questionnaire online by themselves using their own smart device. Study staff made individual appointments to speak with potential participants who were not available for the information sessions. If a potential participant was too busy or refused to participate, an alternate HCP was randomly selected from the roster as a replacement.

### 2.6. Data Analysis

Data were entered, cleaned, and analyzed using the Statistical Package for the Social Sciences (SPSS), version 22.0 (IBM Corporation, Armonk, NY, USA). Descriptive statistics included frequencies, percentages, and means where appropriate.

Odds ratios (OR) were calculated to determine the relationships between the predictor variables (participant characteristics) and the outcome variable (composite variable of negative attitudes toward PLHIV). All predictor variables with significant relationships were included in the binary logistic regression analysis and the results were reported as Adjusted Odds ratios (AOR). A *p*-value < 0.05 was considered statistically significant for all analyses.

### 2.7. Ethical Consideration

The study was approved by the Research Ethics Committee of Faculty of Medicine, Chiang Mai University (Certificate number 177/2019). Participation was voluntary, and all participants provided written informed consent prior to participation.

## 3. Results

### 3.1. Participant Characteristics

Of 3056 participants, 83.3% were female and the average age was 39.2 years old. Most were professional health staff (63.9%), followed by support-clinical staff (20.8%), and support-nonclinical staff (15.0%). The largest group of participants were from the northeastern region (31.1%) while the smallest group were from Bangkok (7.3%). Regarding specialized care for PLHIV, only 21.9% worked at HIV-focused clinics and 21.2% had provided care for more than 20 PLHIV during the last 12 months (Table 1).

### 3.2. Stigmatizing Attitudes toward PLHIV

Eighty-two percent of the respondents had at least one of the four stigmatizing attitudes. When considering each issue individually, the most popular stigmatizing belief was disagreement with “women living with HIV should be allowed to have babies if they wish” (51.9%), followed by agreement with “people get infected with HIV because they engage in irresponsible/immoral behaviors” (48.2%), “people living with HIV should be ashamed about their HIV status” (41.3%), and “most people living with HIV do not care that they could infect other people” (39.3%) (Table 2).

### 3.3. Correlates of Stigmatizing Attitudes

HCP characteristics significantly associated with stigmatizing attitudes were the same in the bivariate and multivariate analyses. These included younger age, being a support-clinical or support-nonclinical staff, working in the central or northeastern region, not working at a HIV-focused clinic, and having a smaller number of PLHIV patients in the last 12 months (Table 3).

## 4. Discussion

In this nationwide probability sampled survey, 82.2% of Thai HCP had stigmatizing attitudes toward PLHIV. This rate seems very high, as participants had to have at least one stigmatizing belief for inclusion in this group, and these results should not be compared directly with the degree or strength of stigmatizing attitudes toward PLHIV as documented in other studies. This composite indicator serves as a reference point for stigmatizing attitudes among Thai HCP towards PLHIV, and can be used to determine long-term trends.

When each stigmatizing attitude is considered separately, “Women living with HIV should not allowed to have babies” was the only item in which more than half of the participants expressed negative attitudes. High levels of stigma regarding the rights of women living with HIV to have children was also found among HCP in India [24]. It was possible that HCP still perceived a high probability of mother to child HIV transmission despite the fact that actual risk in Thailand was very low due to ARV treatment [38]. It is also possible that they may anticipate that children born to HIV-affected families will have difficult lives. “People get infected with HIV because they engage in irresponsible/immoral behaviors” was the second most prevalent belief. This belief is commonly held not only by HCP [19,20,39], but also by the general population [18,40].

HCP whose main responsibility was related to HIV/AIDS, or who provided care to a larger number of PLHIV in the last 12 months, were less likely to have stigmatizing attitudes toward PLHIV. This was in line with studies from Iran, Lao PDR, and Italy, which found that stigmatizing attitudes were lower in HCP experienced in treating HIV/AIDS patients [19,22,23]. In our study, support-clinical and support-nonclinical hospital staff were more likely to have stigmatizing attitudes toward PLHIV as compared to professional staff. Studies from Nigeria and Iran also documented higher prevalence of stigmatizing beliefs among support staff [8,19]. It is likely that professional health staff do not have as many stigmatizing beliefs given that they receive more education and HIV/AIDS training. Increased age was found to be associated with less stigmatizing attitudes toward PLHIV, which was similar to other studies [19,23]. HCP in central and northeastern regions were more likely to have negative attitudes than HCP in Bangkok. This was an interesting finding which needs further investigation to verify the results and to gain a better understanding of this phenomenon.

The current study confirmed the existence of widespread stigmatizing attitudes toward PLHIV among Thai HCP. Currently, Thailand has developed and implemented interventions to address drivers of HIV-related stigma in pilot health facilities using a quality improvement approach [41]. The country also designed an online stigma reduction training program tailored to Thai HCP in the hopes of reaching more staff. These stigma reduction training programs for HCP include a training module addressing stigmatizing attitudes toward PLHIV. The programs are ongoing, and their effectiveness has not yet been established. Future surveillance should explore whether these interventions are effective in reducing stigmatized attitudes among Thai HCP toward PLHIV.

While older professional HCP and staff with more experience taking care of PLHIV have fewer stigmatizing attitudes toward PLHIV, they should not be excluded from participation in the stigma reduction training program. While their level of stigmatizing attitudes may be statistically significantly different from their counterparts, the rate for having at least one stigmatizing attitude was still very high. For example, 70.6% of HCP who worked at HIV-focused clinics had stigmatizing attitudes toward PLHIV as compared to 85.5% of HCP working in other departments. Selected personnel from these groups could be good peer educators or role models for the stigma reduction campaign within their healthcare facilities. Trained popular opinion leaders have been found to be effective in reducing negative attitudes among HCP [42].

The nationwide probability sampling is a strength of this study. Study participants should be a good representation of all Thai HCP. Limitations of the study included potential social desirability bias due to the negative nature of the topic. However, this was minimized by clearly communicating the anonymity of participation and provision of privacy during questionnaire completion. The fact that HIV knowledge and awareness of HIV-related stigma, the other two significant drivers of HIV-related stigma in healthcare settings, have not been studied. Information from this study alone would not be inclusive enough to design an intervention to reduce HIV-related stigma in healthcare settings. We did not record the refusals during data collection. However, the refusal rate was quite low according to feedback received from fieldwork staff. Generalizability of the study results is also limited, as the level of stigmatizing attitudes toward PLHIV and its determinants among HCP vary in different socio-cultural and working environments.

These surveillance surveys yield their full benefits only when conducted regularly using the same methodology and tools. Thailand has planned to conduct this survey every 2 years to document trends. Recommended future studies include HIV knowledge and awareness of HIV-related stigma. Qualitative studies to gain more understanding and identify common root causes of the stigmatizing attitudes are also needed.

## 5. Conclusions

The 2019 national surveillance survey among Thai HCP revealed prevalent stigmatizing attitudes toward PLHIV. HCP who had more work experience especially experience related to PLHIV care were less likely to have stigmatizing attitudes. These personnel could be good peer educators or role models for a stigma reduction campaign within their healthcare facilities.

## Figures and Tables

**Table 1:** Characteristics of Thai Healthcare Personnel (*N* = 3056).

Characteristics	*n* (%)
Gender	
Female	2546 (83.3)
Male	508 (16.6)
Did not answer	3 (0.1)
Age (years) (mean = 39.2, SD = 10.1)	
<30	634 (20.7)
30–39	904 (29.6)
40–49	868 (28.4)
≥50	558 (18.3)
Did not answer	92 (3.0)
Profession	
Health Professional	1952 (63.9)
Support-clinical	635 (20.8)
Support-nonclinical	458 (15.0)
Did not answer	11 (0.4)
Region	
Bangkok	224 (7.3)
Central	886 (29.0)
Northern	494 (16.2)
Northeastern	951 (31.1)
Southern	501 (16.4)
Worked at HIV-focused clinic	
Yes	669 (21.9)
No	2387 (78.1)
Number of PLHIV provided care for during the last 12 months	
0	833 (27.3)
1–4	901 (29.5)
5–20	675 (22.1)
>20	647 (21.2)

PLHIV = people living with HIV.

**Table 2:** Stigmatizing attitudes toward PLHIV by Thai healthcare personnel (*N* = 3056).

Stigmatizing Attitudes	*n* (%)
Women living with HIV should be allowed to have babies if they wish. (disagree)	1586 (51.9)
People get infected with HIV because they engage in irresponsible/immoral behaviors. (agree)	1474 (48.2)
People living with HIV should be ashamed about their HIV status. (agree)	1262 (41.3)
Most people living with HIV do not care that they could infect other people. (agree)	1200 (39.3)
Had at least one stigmatizing attitude	2513 (82.2)

**Table 3:** Factors associated with stigmatizing attitudes toward PLHIV by Thai healthcare personnel (*N* = 3056).

Characteristics	*n*/*N* (%)	OR (95% CI)	AOR (95% CI)
Gender			
Female	2091/2546 (82.1)	ref	a
Male	421/508 (82.9)	1.05 (0.82–1.36)	
Age (years)			
<30	543/634 (85.6)	1.65 (1.22–2.23) *	1.60 (1.18–2.18) *
30–39	765/904 (84.6)	1.52 (1.16–2.00) *	1.60 (1.21–2.12) *
40–49	693/868 (79.8)	1.10 (0.85–1.82)	1.16 (0.89–1.53)
≥50	437/558 (78.3)	ref	ref
Profession			
Health Professional	1543/1952 (79.0)	ref	ref
Support-clinical	556/635 (87.6)	1.87 (1.44–2.42) *	1.89 (1.44–2.49) *
Support-nonclinical	403/458 (88.0)	1.94 (1.44–2.63) *	1.71 (1.24–2.36) *
Region			
Bangkok	169/224 (75.4)	ref	ref
Central	759/886 (85.7)	1.95 (1.36–2.78) *	1.84 (1.27–2.68) *
Northern	398/494 (80.6)	1.35 (0.93–1.97)	1.26 (0.85–1.87)
Northeastern	789/951 (83.0)	1.59 (1.12–2.25) *	1.63 (1.13–2.34) *
Southern	398/501 (79.4)	1.26 (0.87–1.83)	1.32 (0.89–1.95)
Currently working at HIV-focused clinics			
Yes	472/669 (70.6)	ref	ref
No	2041/2387 (85.5)	2.46 (2.01–3.01) *	1.97 (1.57–2.48) *
Number of PLHIV provided care for during the last 12 months			
0	716/833 (86.0)	2.43 (1.88–3.15) *	1.62 (1.21–2.17) *
1–4	767/901 (85.1)	2.28 (1.77–2.92) *	1.94 (1.48–2.54) *
5–20	567/675 (84.0)	2.09 (1.60–2.73) *	1.87 (1.40–2.45) *
>20	463/647 (71.6)	ref	ref

“stigmatizing attitudes toward PLHIV” was the main dependent variable and was defined as “reporting. at least one stigmatizing attitude toward PLHIV”. a = not included in multivariate analysis. * = statistically significant result. OR = odds ratio. AOR = adjusted odds ratio.

## Data Availability

The data presented in this study are openly available in FigShare at https://figshare.com/articles/dataset/Thailand_healthcare_HIV_stigma_survey_2019/14816271. doi: 10.6084/m9.figshare.14816271 (accessed on 21 June 2021).
